# P-1284. Impact of Timing of Measles Vaccine Doses on IgG Levels

**DOI:** 10.1093/ofid/ofae631.1465

**Published:** 2025-01-29

**Authors:** Évrard Pérès, Zineb Laghdir, Sophie Boissonneault, Annie St-Pierre, Émilie Vallières, Caroline Quach

**Affiliations:** Centre hospitalier universitaire Sainte-Justine, Montréal, Quebec, Canada; Centre hospitalier universitaire Sainte-Justine, Montréal, Quebec, Canada; Centre hospitalier universitaire Sainte-Justine, Montréal, Quebec, Canada; Centre hospitalier universitaire Sainte-Justine, Montréal, Quebec, Canada; Centre hospitalier universitaire Sainte-Justine, Montréal, Quebec, Canada; University of Montreal/CHU Sainte-Justine, Montreal, Quebec, Canada

## Abstract

**Background:**

Although measles was eliminated from Canada, recent outbreaks worldwide are increasing the risk of importation with local transmission. Healthcare workers (HCWs) are known to be at higher risk of measles, compared to the general population. Recent measles breakthrough infection in immune individuals have raised several questions about levels of immunity. We examined the proportion of HCWs who were considered seropositive for measles and evaluated factors associated with lower IgG levels.

**Methods:**

Measles IgG were measured in a convenient sample of HCWs from the *Centre hospitalier universitaire Sainte-Justine* (CHUSJ), a tertiary-care hospital, located in Montreal, Canada, in the context of an outbreak and the need to rapidly assess our workforce protection. Data from the Occupational Health clinic was used (age, vaccination history, sex). Sera were tested using LIAISON® Measles IgG (Diasorin) for quantitative measurement of measles-specific IgG. IgG levels > 16.5 IU/L were considered protective. Data were compared using the Welch’s *t*-test on Microsoft Excel.

**Results:**

A total of 114 HCWs were tested. Of the 42 individuals born before 1970 without vaccination, 92.9% were considered protected with a mean IgG level of 239.07 IU/L (SD - 103). A total of 45 HCWs were born in 1970 or thereafter; of those 32 received two doses of vaccine, with a first dose received between 12 and 15 months of age. HCWs (n = 8) who received their second dose between 17 and up to 21 months of age had mean IgG titers of 71.38 IU/L (SD - 104). In comparison, the 24 HCWs who received their second dose after 21 months of age, at a mean age of 142.43 months (range [28,393]), had a mean IgG level of 177.12 IU/L (p = 0.03).

**Conclusion:**

These results suggest that the second dose of measles vaccine administered at a later age is associated with a higher IgG level. The clinical impact of these higher IgG levels remains to be explored.

**Disclosures:**

**All Authors**: No reported disclosures

